# Supplementary material for: High Mortality and Graft Loss after Infective Endocarditis in Kidney Transplant Recipients: A Case-Controlled Study from Two Centers
Source: Pathogens. 2021 Aug 13;10(8):1023. doi: 10.3390/pathogens10081023 (PMC8397984; doi:10.3390/pathogens10081023)
Supplement: Supplementary file 1 [file pathogens-10-01023-s001.zip › pathogens-1282758-supplementary.pdf]

Supplementary tables

Supplementary Table S1. Characteristics of the patients who suffered an IE complicated by vascular embolism.

Characteristics	N = 6, n (%), or Mean ± SD
**Valve**	
Native	6 (100)
Aortic IE	3 (50)
Mitral IE	3 (50)
Ring abscess and/or severe valve leakage	3 (50)
**Microbiology**	
Enterococci	2 (33.3)
Staphylococcus aureus	2 (33.3)
Streptococcus gallolyticus	1 (16.7)
Escherichia coli	1 (16.7)
**Treatment**	
Antibiotic treatment duration (weeks)	6.0 ± 3.5
Aminoglycoside use	6 (100)
Indication for surgery	5 (83.3)
Surgery	1 (16.7)
One year mortality	3 (50)

IE: infective endocarditis.

Supplementary Table S2. Characteristics of the cases of IE complicated by death or graft loss (without death over the study period).

Characteristics	IE Complicated by Death N = 8 n (%) or Mean ± SD	IE Complicated by Graft Loss and Not Death N = 5 n (%) or Mean ± SD
**Valve**		
Native	8 (87.5)	3 (60)
Prosthetic	1 (12.5)	2 (40)
Aortic IE	4 (50)	2 (40)
Mitral IE	4 (50)	1 (20)
Mitral and aortic IE	0 (0)	2 (40)
Ring abscess and/or severe valve leakage	4 (50)	1 (20)
**Microbiology**		
Enterococci	4 (50)	2 (40)
Staphylococcus aureus	3 (37.5)	0 (0)
Escherichia coli	1 (10)	(0)
Staphylococcus epidermidis	0 (0)	2 (40)
No documentation	0 (0)	1 (20)
**Treatment**		
Antibiotic treatment duration (weeks)	NA *	6 ± 0
Aminoglycoside use	7 (87.5)	2 (40)
Indication for surgery	6 (75)	1 (20)
Surgery	1 (12.5)	0 (0)

* Not Applicable due to early death.
